# Machine learning models identify micronutrient intake as predictors of undiagnosed hypertension among rural community-dwelling older adults in Thailand: a cross-sectional study

**DOI:** 10.3389/fnut.2024.1411363

**Published:** 2024-07-16

**Authors:** Niruwan Turnbull, Le Ke Nghiep, Aree Butsorn, Anuwat Khotprom, Kukiat Tudpor

**Affiliations:** ^1^Faculty of Public Health, Mahasarakham University, Maha Sarakham, Thailand; ^2^Public Health and Environmental Policy in Southeast Asia Research Cluster (PHEP-SEA), Mahasarakham University, Maha Sarakham, Thailand; ^3^Vinh Long Department of Health, Vinh Long, Vietnam; ^4^College of Medicine and Public Health, Ubon Ratchathani University, Ubon Ratchathani, Thailand

**Keywords:** undiagnosed hypertension, older adults, machine learning, dietary intake, community health

## Abstract

**Objective:**

To develop a predictive model for undiagnosed hypertension (UHTN) in older adults based on five modifiable factors [eating behaviors, emotion, exercise, stopping smoking, and stopping drinking alcohol (3E2S) using machine learning (ML) algorithms.

**Methods:**

The supervised ML models [random forest (RF), support vector machine (SVM), and extreme gradient boosting (XGB)] with SHapley Additive exPlanations (SHAP) prioritization and conventional statistics (χ^2^ and binary logistic regression) were employed to predict UHTN from 5,288 health records of older adults from ten primary care hospitals in Thailand.

**Results:**

The χ^2^ analyses showed that age and eating behavior were the predicting features of UHTN occurrence. The binary logistic regression revealed that taking food supplements/vitamins, using seasoning powder, and eating bean products were related to normotensive and hypertensive classifications. The RF, XGB, and SVM accuracy were 0.90, 0.89, and 0.57, respectively. The SHAP identified the importance of salt intake and food/vitamin supplements. Vitamin B6, B12, and selenium in the UHTN were lower than in the normotensive group.

**Conclusion:**

ML indicates that salt intake, soybean consumption, and food/vitamin supplements are primary factors for UHTN classification in older adults.

## Introduction

Hypertension is acknowledged as a significant risk factor for stroke and cardiovascular diseases worldwide (1). Furthermore, hypertension accounts for more than one-fifth of all cardiovascular disorders and is the primary risk factor for approximately 10 million deaths and 200 million disabilities globally. It is recommended that early diagnosis of hypertension can lead to timely treatment and risk factor management, reducing associated morbidity (2). Systolic hypertension is defined as a multifaceted chronic condition with systolic blood pressure (SBP) of ≥140 mmHg, under European Society of Cardiology/European Society of Hypertension (ESC/ESH) recommendations (3, 4). A condition with SBP of ≥140 mmHg without a history of hypertension diagnosis from a medical practitioner and antihypertensive therapy is considered undiagnosed hypertension (UHTN) (5). Even though the global prevalence of UHTN is estimated at 22%, it varies among different populations and geographical areas (6, 7). In India, approximately 40% of older adults have UHTN, with a higher rate in rural areas (8). Meanwhile, in Ethiopia, the prevalence of UHTN is estimated to be 30% (9). In contrast, in the United States of America, the UHTN has been reportedly between 4 and 7% (10). According to the latest health survey (sixth National Health Examination Survey, NHES VI) conducted in Thailand in 2019, the proportion of UHTN was 40.5 and 57.0% among male and female populations, respectively (11). From a clinical perspective, early-diagnosed patients may benefit from prompt treatment considerations due to chronically high blood pressure, which can provide them an opportunity to modify the causes of hypertension (12).

The etiology of hypertension depends on non-modifiable (such as sex, age, and genetics) and modifiable factors (such as consumption behaviors, psychological stress, physical activity, tobacco use, and alcohol consumption) (13). Previous studies suggest that by monitoring and controlling the modifiable risk factors, up to 90% of hypertension-associated cardiovascular diseases (CVDs) can be prevented. Thus, correctly identifying people with these risk factors and those who are vulnerable is an essential first step in controlling CVDs. In Thailand, five modifiable factors – 3E (eating behaviors, emotion, exercise) and 2S (stop smoking and stop drinking alcohol) have been focused and applied to improve clinical outcomes in hypertensive patients. A previous study demonstrated that combining 3E2S-related behavioral changes with regularly taking antihypertensive medications and recognizing acute episodes was effective in controlling blood pressure in primary care settings (14). Recently, the 3E2S principle has been utilized to manage other chronic non-communicable diseases (NCDs), such as stroke and coronary artery diseases in older adults (15).

In public health, artificial intelligence (AI) and ML technology have contributed to significant advances in predicting epidemics of diseases (16). For example, an ML model (RF algorithm) can predict body mass index from the voice features of the participants (17). Recently, hypertension prediction models were developed and compared using three supervised machine learning algorithms: decision tree, logistic regression, and RF. Modifiable factors included age, sex, education level, employment, tobacco use, physical activity, and adequate fruit and vegetable consumption were identified as predictors (18). In addition, SHAP, a technique that identifies and prioritizes characteristics of combined categorization and event prediction with ML models, signified that age and serum biomarkers (alkaline phosphatase and triglycerides) were important features for predicting the risk of hypertension (19). These reports suggest alternative methods to predict UHTN besides the conventional statistical analyses. Therefore, this present study aimed to identify the relationship between UHTN and 3E2S among older adults in Northeastern Thailand using conventional statistics (χ^2^ analysis and binary logistic regression) and supervised ML models RF, SVM, XGB, and SHAP.

## Materials and methods

### Research design and participants

This descriptive cross-sectional study was performed on 5,288 health records of non-hypertensive older adults from ten primary care hospitals in Northeastern Thailand. This subproject was a part of the Ministry of Digital Economy and Society’s Office of the National Digital Economy and Society Commission project, “Development of Mobile Application of Database of Older Persons Using Geographic Information System (GIS) to Detect and Analyze Risks of Chronic Diseases, Quality of Life, and Mental Illness by Village Health Volunteers in 7^th^ Regional Health Office Territory.” Each protocol followed the Declaration of Helsinki and was authorized by the Maha Sarakham Provincial Public Health Office’s Ethical Review Committee for Human Research (No. 6/2564). The study complies with the recommendations of the Committee on Publication Ethics (COPE) and the International Committee of Medical Journal Editors (ICMJE) for ethics. Each participant provided written informed permission on an official document. The inclusion criteria were age ≥ 60, not being diagnosed hypertensive, and a household registered with informed consent in four provinces. Older persons not present in person for the study were omitted. The participants with SBP < 140 mmHg were normotensive, while those ≥140 mmHg were UHTN based on the ICD-10 guideline (3, 4).

### Data collection tools

There were two components to the data collection instruments used in the present study. Initially, information on sex, age, body mass index (BMI), marital status, and educational attainment was gathered. Because of the potential risks associated with disease, weakness, hospitalization, and falls, 75 was selected as the cut-off age (20). In the second part, 3E2S data were collected. A structured questionnaire for consumption behaviors was developed and had 16 items with 4 levels of choices (never = 1, sometimes = 2, frequently = 3, and always = 4). Therefore, the possible total score was 64. The consumption behaviors were divided into 3 groups (≤ 21.5 = very poor, > 21.5 to 43 = poor, and 44 to 64 = good). Therefore, a score of 44 was used as a cut-off point between good and poor consumption behaviors. A structured questionnaire for hypertension-related consumption behaviors had 14 items of yes-no questions, as shown in Table 1. A questionnaire on psychological stress was used as previously described (21). The physical activity score was 1 for those performing at least 150 min/week of moderate-intense exercise and 0 for those not following the WHO guidelines (22). Likewise, never or stop smoking tobacco and drinking alcohol were scored 1, while current smoking and drinking were scored 0. Three public health, psychiatry, and nursing specialists approved the instrument’s content validity. The index of item-objective congruence (IOC) has verified the content validity of the consumption behavior, hypertension-related, and psychological stress questionnaires. For the surveys, only items with IOC scores ≥0.5 were acceptable. The questionnaire included items that met the acceptable Cronbach’s α ≥ 0.7 thresholds. The team of trained village health volunteers, nurses, public health practitioners, and physical therapists compiled the primary data in the area between April and August of 2021 through mobile phone apps and online forms.

**Table 1:** Binary logistic analysis of eating behaviors on a hypertensive class prediction.

Feature of eating behavior	Hypertensive class		
	OR	95% CI	*p*-value
1. Having breakfast			0.057
No	1.00		
Yes	7.21	0.94–55.11	
2. Taking food supplements/vitamins			0.045^*^
No	1.00		
Yes	0.83	0.69–0.99	
3. Taking a meal high in fat			0.296
No	1.00		
Yes	1.23	0.83–1.82	
4. Using table fish sauce with chili			0.720
No	1.00		
Yes	1.04	0.83–1.30	
5. Using seasoning powder			0.007^*^
No	1.00		
Yes	1.23	1.06–1.42	
6. Eating dessert after meal			0.159
No	1.00		
Yes	0.82	0.62–1.08	
7. Eating spicy food			0.273
No	1.00		
Yes	1.28	0.82–2.00	
8. Eating at least half a kilo of fruits or vegetables a day			0.451
No	1.00		
Yes	0.91	0.71–1.17	
9. Eating the hard-to-digest or fatty food			0.970
No	1.00		
Yes	0.99	0.61–1.60	
10. Eating grilled food			0.597
No	1.00		
Yes	0.93	0.69–1.24	
11. Eating fish or chicken			0.702
No	1.00		
Yes	0.90	0.51–1.59	
12. Eating or drinking bean products			0.015^*^
No	1.00		
Yes	0.61	0.42–0.91	
13. Eating convenience food			0.382
No	1.00		
Yes	0.88	0.67–1.67	
14. Eating leftover			0.508
No	1.00		
Yes	0.85	0.53–1.37	

OR, odds ratio; ^*^p-value < 0.05. The normotensive class was set as a reference.

### Calculation of micronutrient intake

Using the software program INMUCAL, developed by Mahidol University, Thailand, the intake of micronutrients was determined based on the 24-h food record [Institute of Nutrition (23)]. According to the manufacturer, the program is standard for estimating the intake of macro- and micronutrients in Thai food. The consumption levels of ten vitamins (vitamin A, retinol, β-carotene, vitamin B1, vitamin B2, vitamin B3, vitamin B6, vitamin B12, vitamin C, and vitamin E) and nine minerals (calcium, copper, iron, magnesium, phosphorus, potassium, selenium, sodium, and zinc) were determined. Sample size (*n*) was estimated using the formula as given: $n=\frac{Nz^{2}pq}{+d^{2}\left(N-1\right)+z^{2}pq\;}$ (24), where *N* represents the total number of the older adult population, which was 5,288, *z* = percentiles of the standard normal distribution corresponding to a 95% confidence level, which is 1.96, *p* = proportion of the older adults with undiagnosed hypertension, which was assumed to be 0.17, *q* = 1 − *p*, and *d* denotes margin of error = 5 at 95% confidence level. Therefore, using the formula, *n* = 5,288(1.96)^2^ × 0.17 × 0.83/(0.05)^2^(5,288–1) + (1.96)^2^ × 0.17 × 0.83 = 08.32 ~ 209. The sample has been randomly selected from the total number of the older adult population.

### Machine learning models for hypertensive risk prediction

We used supervised machine learning techniques to analyze the relationship between the datasets of SBP groups and categorical behavioral data. Regarding feature selection strategies for machine learning, the features with *p*-value <0.05 in χ^2^ and binary logistic analysis were selected. Demographic attributes (age and education) and nominal data attributes (sex, BMI, marital status, stress levels, and consumption behaviors) were then transformed into categorical data. This shift allows for a more applicable exploration of the relationship between these variables and SBP status, indicating the inherent order and significance within these categories. In the dataset, there were 894 hypertensive cases and 4,394 normotensive individuals (control). This class imbalance could lead to a biased model prioritizing the majority class, which could not be generalized for the classification task. To mitigate this, SMOTE was employed to balance the class distribution by generating synthetic examples for the minority class. In this case, it involved creating synthetic instances for the hypertensive class. The algorithms applied were (1) the selection of a data point from the minority class (hypertensive) and the identification of its k nearest neighbors in feature space, (2) the synthetic generation of data instances by linearly interpolating between the chosen data point and one of its nearest neighbors, and (3) repetition of (1) and (2) until the desired over-sampling factor was met. Once the dataset was balanced, with an increased number of synthetic instances for the hypertensive class, we performed to make the class distribution more equitable. After the data transformation process, classification using machine learning algorithms was performed. The initial classification process was derived through a mapping function f:X→y, where X represents the transformed input data and denotes the class labels, categorized as 1 for hypertensive and 0 for normotensive. The RF algorithm was first introduced for the initial classification of this classification problem. All variables were transformed into nominal variables by one-hot encoding.

The RF constructs an ensemble of decision trees during training, with each tree making predictions based on a subset of the data and features. Decision-making is performed by aggregating predictions from individual trees, and the final class is determined by the mode of these predictions (majority vote schemes). Ordinal variables were naturally handled by decision trees through threshold-based splits, while nominal variables were encoded for effective processing. In addition to RF, the SVM was exploited by seeking the optimal hyperplane that linearly separates the data into distinct classes. For binary classification, the hyperplane equation was represented as w⋅x+b=0 where w was the weight vector, and b was the bias term. The decision-making function was expressed as $f\left(x\right)=\text{sign}\;\left(\sum_{i=1}^{n}\alpha_{i}y_{i}K\left(x,x_{i}\right)+b\right)$ where $\alpha_{i}$ were coefficients, $y_{i}$ were class labels, $x_{i}$ were training samples, K was the kernel function, and b was the bias term. Additionally, the XGB algorithm was operated by iteratively constructing a series of decision trees, each addressing the weaknesses of its predecessors. The model was aimed to optimize a loss function by minimizing errors and penalizing misclassifications. Feature importance is inherent in XGB, providing a natural ranking of variables based on their contribution to the model’s performance.

**Figure 1:**
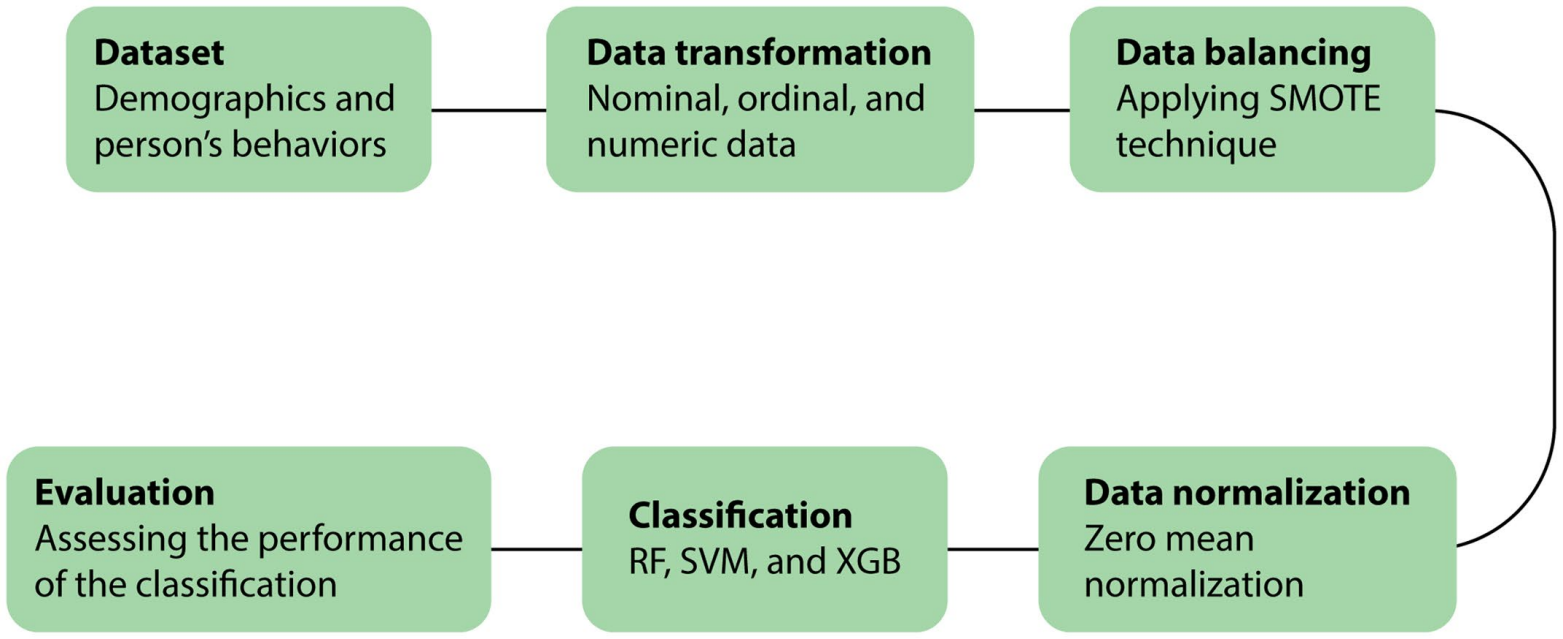
The process of the classification.

Machine learning algorithms were run on Python 3.10.12 in Google Colab. Hyperparameter tuning was used to optimize the RF using max_depth: [5,12,14,16,18,20], min_samples_leaf: [6,8,10,12,14,16], n_estimators: [10,25,30,50,70,100]. This setting prevented overfitting and reduced the performance of the prediction model. The case/control proportion was 894 hypertensive cases and 4,394 normotensive individuals (control) with 5-fold cross-validation (Supplementary data S2). For SVM, C: [0.1, 1, 10, 100, 1,000], gamma: [1, 0.1, 0.01, 0.001, 0.0001], kernel: [‘linear’, ‘poly’, ‘rbf’, ‘sigmoid’] were employed. Parameters max_depth, min_samples_leaf, and n_estimators in this range can prevent overfitting, which can reduce the performance of the prediction model. The ML process is depicted in Figure 1.

SHAP (Shapley Additive Explanations) explains model predictions that employ Shapley values from cooperative game theory. In this context, each feature is considered a “player” in the game, with the model’s prediction serving as the game’s “payout.” The Shapley values show how to evenly allocate the game’s payout to each player based on their contribution. Regarding model analysis, these numbers indicate how much each feature impacts the model’s prediction (25). The principle of Shapley values can be efficiently applied to an RF model using the internal workings of the decision trees to compute the Shapley values – path traversal exploring the different paths within the tree chosen by each sample, contribution calculation calculating the contribution of each feature along each path, and aggregation (aggregating the contributions from all paths to obtain the final Shapley values) (26). SHAP consists of 5 steps – (1) local explanations with SHAP values (SHAP values quantify the contribution of each feature to the prediction for a particular instant based on values from cooperative game theory, adapted for machine learning contexts), (2) aggregation to understand global behavior, (3) importance ranking (features with higher average SHAP values are considered more important as they have a more significant impact on model predictions across the dataset), (4) visualization and interpretation (summary dot plots) that show the importance of each feature, and (5) quantifying impact, which helps in understanding the relative influence of different features on the model’s output (27). The practical method for Python users can be found at the SHAP documentation website: https://shap.readthedocs.io and https://pypi.org/project/shap/.

### Statistical analyses

Continuous data are expressed as mean and standard deviation. The normality of sample data was tested using the Kolmogorov–Smirnov test. Two means of independent non-normally distributed samples were analyzed using the Mann–Whitney U test. Categorical data are expressed as frequency and percentage. For the univariate analysis, relationships between SBP, demographic variables, and 3E2E variables were evaluated and compared using χ^2^ tests. The results showed that there was minimal multicollinearity among the variables. Binary logistic analysis was performed to investigate the simultaneous effect of multiple factors on a dichotomous outcome using SPSS software version 18.

## Results

The socio-demographic data of 5,288 older adults from 8 subdistricts are shown in Table 2. Most participants were females with a mean age of ≤75 years old, had normal weight, were married, and had a primary school education level (Table 2). Age groups were associated with blood pressure classes (χ^2^ = 4.62, *p* = 0.03).

**Table 2:** Association between demographic characteristics with blood pressure in older adults (n = 5,288).

Feature	Class		χ^2^	*p*-value	Contingency coefficient
	Normotensive	Hypertensive			
Sex			2.23	0.13	0.021
Male	1,834 (41.74)	349 (39.04)			
Female	2,560 (58.26)	545 (60.96)			
Age (years)			4.62	0.03*	0.032
60–75	3,442 (78.33)	671 (75.06)			
≥ 76	952 (21.67)	223 (24.94)			
BMI (kg/m^2^)			9.46	0.38	0.002
Underweight (≤ 18.4)	426 (9.95)	100 (11.55)			
Normal weight (18.5–22.9)	1,929 (45.07)	369 (42.61)			
Overweight (23–24.9)	891 (20.82)	179 (20.67)			
Obese (≥ 25)	1,034 (24.16)	218 (25.17)			
Marital status			0.66	0.41	−0.011
Single/divorced/widowed**/**separated	1,564 (35.59)	331 (37.03)			
Married	2,830 (64.41)	563 (62.97)			
Education			6.32	0.09	0.003
Unattended school	152 (3.46)	19 (2.13)			
Primary school (grades 1–6)	3,937 (89.60)	824 (92.17)			
Secondary/high school (grades 7–12)	214 (4.87)	36 (4.02)			
Diploma or higher	91 (2.07)	15 (1.68)			

Contingency coefficient: Phi-coefficient for 2×2 tables; Gamma for ordinal tables. ^*^p-value < 0.05.

In the next section, we investigated an association between the 3E2S factors and blood pressure classes. Results showed that only the eating behavior levels were associated with the blood pressure classes (χ^2^ = 10.65, *p* = 0.001) (Table 3).

**Table 3:** Association between 3E2S factors with blood pressure in older adults.

Feature	Class		χ^2^	p-value	Contingency coefficient
	Normotensive	Hypertensive			
Eating behavior level			10.65	0.001*	0.045
Poor	3,453 (78.58)	658 (73.60)			
Good	941 (21.42)	236 (26.40)			
Emotional stress level			0.30	0.86	0.012
Mild	2,527 (57.51)	510 (57.05)			
Moderate	1,598 (36.37)	325 (36.35)			
High	269 (6.12)	59 (6.60)			
Exercise level			0.52	0.46	0.010
< 150 min/week	822 (18.71)	158 (17.67)			
≥150 min/week	3,572 (81.29)	736 (82.33)			
Alcohol drinking			0.76	0.38	−0.012
Not drinking	3,502 (79.70)	724 (80.98)			
Drinking	892 (20.30)	170 (19.02)			
Smoking			1.36	0.24	−0.016
Not smoking	3,882 (88.35)	802 (89.71)			
Smoking	512 (11.65)	92 (10.29)			

Contingency coefficient: Phi-coefficient for 2×2 tables; Gamma for ordinal tables. ^*^p-value < 0.05.

An analysis with the binary logistic analysis also depicted an association between three items of eating behaviors (taking food supplements/vitamins, using seasoning powder, and eating or drinking bean products) and hypertensive class (OR = 0.83, *p* = 0.045; OR = 1.23, *p* = 0.007, and OR = 0.61, *p* = 0.015, respectively) (Table 1).

**Figure 2:**
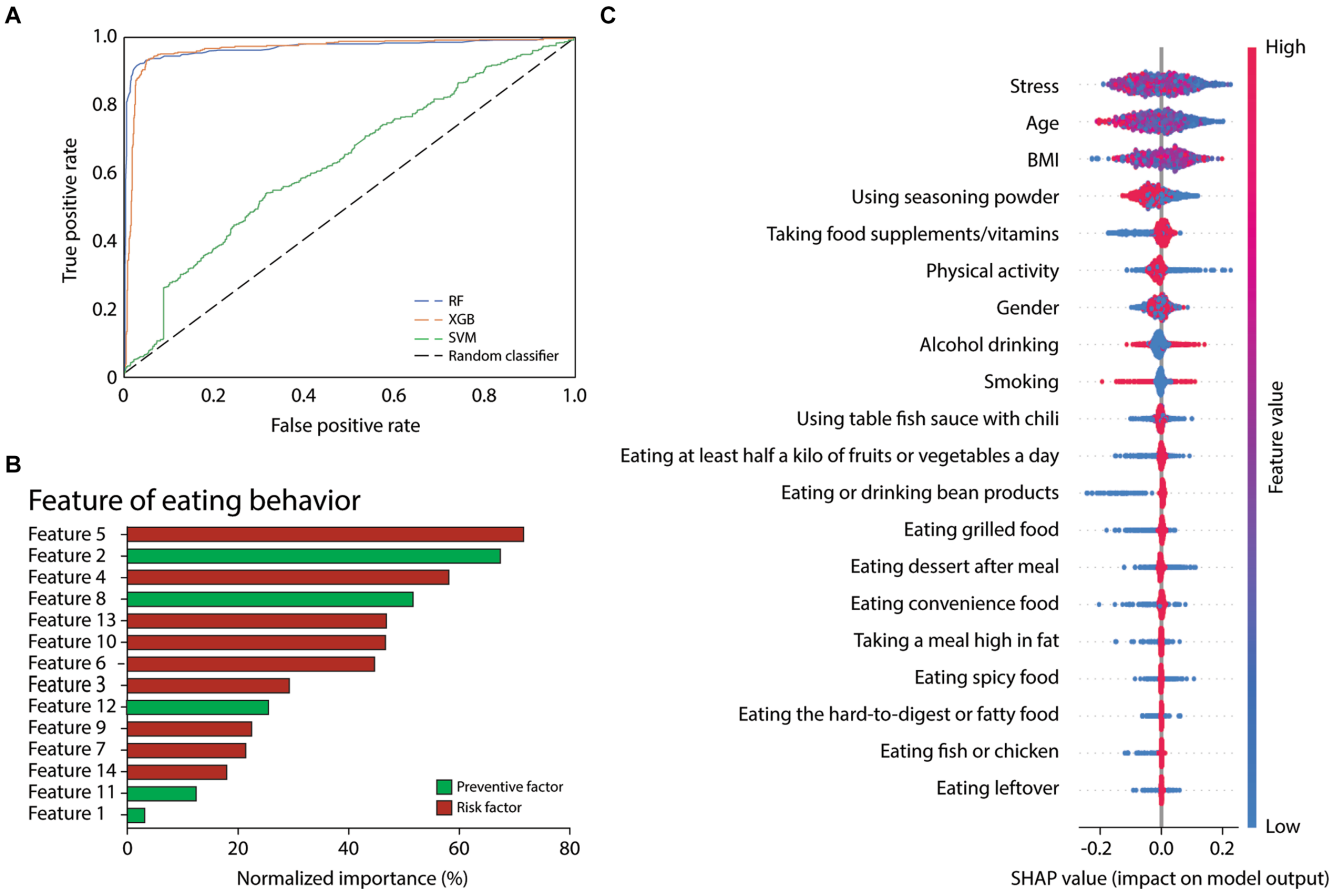
**(A)** ROC curves of the cross-validated RF, XGB, and SVM classification on test data. **(B)** Normalized importance plot of the features of eating behaviors (from Table 3) according to the ROC curve of the cross-validated RF classification. **(C)** SHAP values of the feature impact on the model’s prediction of normotension.

We implemented the holdout method in the machine learning setting and process, allocating 20% of the dataset as test data (1,058 instances). Before the training phase, the dataset underwent a preprocessing step to achieve a balanced distribution. Applying SMOTE, as previously explained, we mitigated class imbalance by down-sampling the majority class and up-sampling the minority class. Consequently, we obtained a dataset comprising 7,022 instances evenly distributed between the positive and negative classes (3,511 instances each). Three prominent machine learning algorithms – RF, XGB, and SVM – were employed as learning models for benchmarking purposes. Results showed that the accuracy of the receiver operating characteristic (ROC) curves from RF, XGB, and SVM were 0.90, 0.89, and 0.57, respectively (Figure 2A). According to the ROC curve of the cross-validated RF classification, the feature importance plot showed that the normalized importance levels of using seasoning powder, taking food supplements/vitamins, using table fish sauce with chili, and eating at least half a kilo of fruits or vegetables a day were 71.7, 67.1, 57.8, and 51.3%, respectively. The importance of the rest of the features was less than 50% (Figure 2B). In addition, SHAP (SHapley Additive exPlanations) values showed that using seasoning powder had a high negative impact, while taking food supplements/vitamins had a high positive impact on the model outputs (Figure 2C).

**Figure 3:**
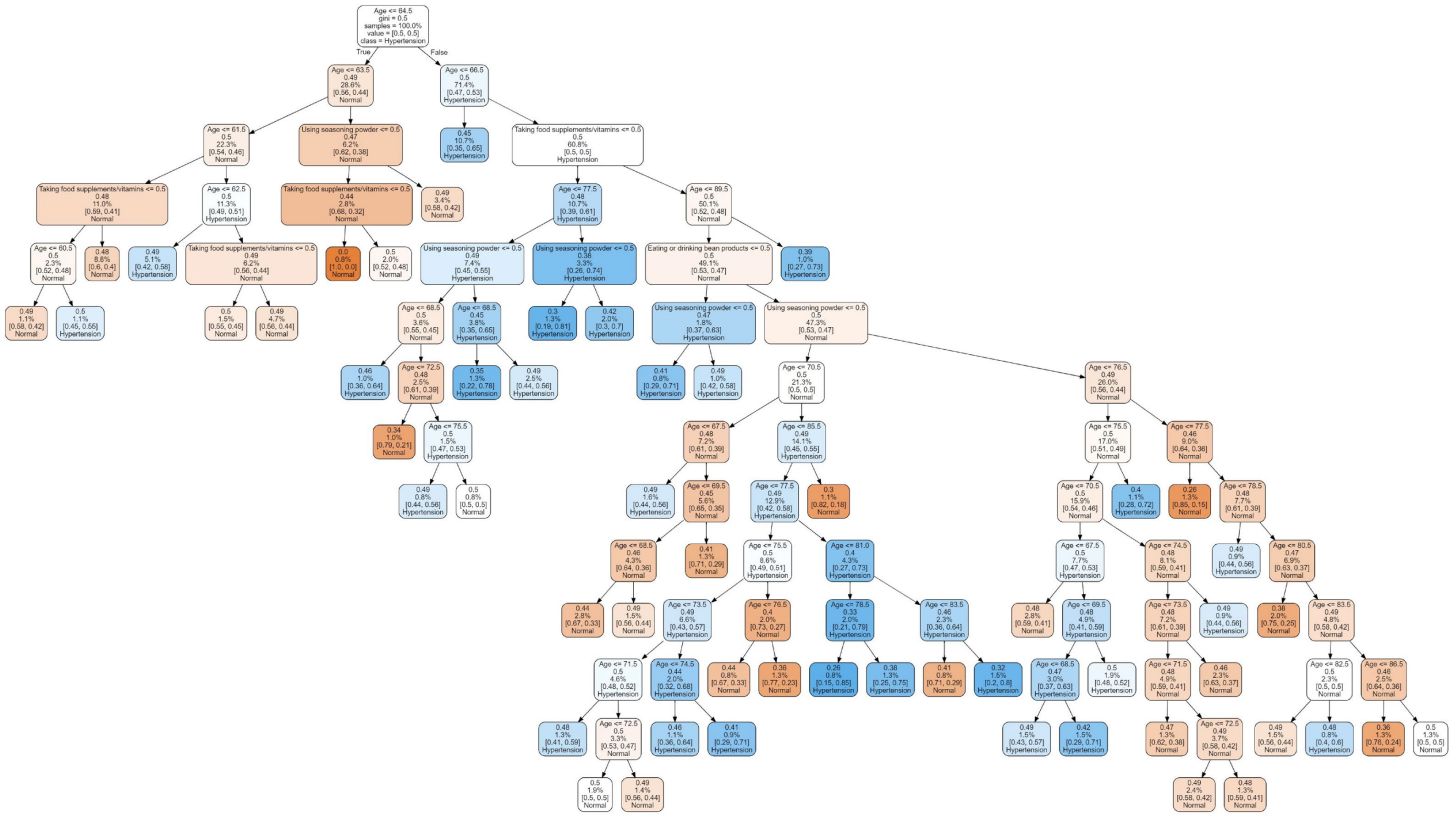
A representative of decision tree predictive model.

Based on our findings that age, eating, and behaviors (taking food supplements/vitamins, using seasoning powder, and eating or drinking bean products) were associated with hypertensive class and that RF was the most suitable predictive ML model, we developed a predictive model using the decision tree method. The decision tree predictive model was built following a public protocol, which can be found here: https://github.com/topics/decision-tree-model. The model contains fifty decision trees, a representative tree depicted in Figure 3.

Using the Mann–Whitney U test, we analyzed the differences in micronutrient consumption levels between the normotensive and hypertensive groups. Results showed that vitamin B6, vitamin B12, and selenium intakes in the hypertensive group were significantly lower than in the normotensive group (*p* = 0.012, 0.036, and 0.011, respectively, Table 4). Raw data are in Supplementary data S1.

**Table 4:** Independent samples Mann–Whitney U tests of macronutrient and micronutrient consumption levels (*n* = 209).

Feature	Class			
	Normotensive	Hypertensive	U statistic	*p*-value
	(*n* = 104)	(*n* = 105)		
Macronutrient				
Carbohydrate (g)	178.0 ± 8.2	183.1 ± 7.7	5713.0	0.719
Protein (g)	38.3 ± 1.6	47.6 ± 4.0	6025.0	0.902
Total fat (g)	20.2 ± 1.7	17.3 ± 1.5	4916.5	0.107
Saturated fat (g)	5.5 ± 0.6	5.2 ± 0.5	5262.0	0.326
Cholesterol (mg)	149.6 ± 15.8	143.0 ± 19.0	5147.5	0.238
Vitamin				
Vitamin A (retinoic activity equivalent)	142.90 ± 20.43	131.50 ± 26.11	5022.0	0.315
Retinol (μg)	75.84 ± 15.11	71.59 ± 22.37	5075.0	0.371
β-carotene (μg)	771.63 ± 157.24	554.63 ± 72.83	5449.5	0.980
Vitamin B1 (mg)	1.63 ± 0.66	0.86 ± 0.14	5165.0	0.421
Vitamin B2 (mg)	0.79 ± 0.05	0.76 ± 0.06	5301.0	0.773
Vitamin B3 (niacin, mg)	11.11 ± 0.55	12.38 ± 0.65	6183.0	0.098
Vitamin B6 (mg)	0.31 ± 0.06	0.25 ± 0.06	4468.5	0.012*
Vitamin B12 (μg)	0.48 ± 0.08	0.37 ± 0.07	4777.5	0.036*
Vitamin C (mg)	62.08 ± 10.73	42.87 ± 9.08	5016.0	0.312
Vitamin E (mg)	0.92 ± 0.21	0.68 ± 0.12	4805.5	0.092
Mineral				
Calcium (mg)	222.63 ± 19.00	236.87 ± 18.72	5708.0	0.572
Copper (mg)	0.52 ± 0.02	0.54 ± 0.02	5782.0	0.423
Iron (mg)	6.82 ± 0.38	7.10 ± 0.31	6086.0	0.148
Magnesium (mg)	31.04 ± 2.45	33.75 ± 3.27	5326.5	0.756
Phosphorus (mg)	373.96 ± 17.15	453.27 ± 36.78	5676.0	0.620
Potassium (mg)	937.28 ± 48.41	960.16 ± 70.07	5079.5	0.383
Selenium (μg)	22.06 ± 3.03	15.36 ± 2.99	4325.0	0.011*
Sodium (mg)	2169.63 ± 193.38	2017.06 ± 134.62	5175.5	0.516
Zinc (mg)	4.15 ± 0.20	4.37 ± 0.19	5938.0	0.279

Data are expressed as mean ± standard error of the mean. ^*^p-value < 0.05.

## Discussion

This study shows that consumption behaviors are predictors that classify blood pressure as normotension and hypertension in community-dwelling older adults. The following findings support our statement. First, among 3E2S, only consumption behaviors are significantly associated with the difference in systolic blood pressure. Secondly, the importance of predicted features (> 50% normalized importance) from ML overlapped with ones predicted by binary logistic regression – using seasoning powder as the risk factor and taking food supplements/vitamins as the prevention of UHTN. In addition, the ML also predicted that using table fish sauce with chili while eating at least half a kilo of fruits or vegetables a day prevented UHTN.

Risk and preventive factors for hypertension are different and overlap among countries. In the United Kingdom, the National Institute for Health and Clinical Excellence (NICE) has developed a practical clinical guideline on hypertension that encourages healthy, calorie-controlled diets, regular aerobic exercise, and reducing alcohol, salt, and tobacco use in overweight individuals with elevated blood pressure (28). In Cameroon, the prevalence of high blood pressure in adults was 19.8%. Age, alcohol use, and a sedentary lifestyle were found to be separate causes of hypertension (29). In the Chinese population, HTN incidence was 28%. Risk variables for hypertension were body mass index, level of physical activity, use of alcohol and tobacco, once-weekly meat consumption, family history, and daily salt intake (30). A study in Bangladesh showed that four primary modifiable risk factors for hypertension are obesity, dyslipidemia, tobacco use, and excessive salt intake (31). Antihypertensive medications and these factors work together to prevent and treat hypertension. In India, after controlling for other risk variables, it was discovered that diabetes, smoking, obesity, and inactivity were all substantial risk factors for hypertension (32). The estimated prevalence of hypertension in Southeast Asians was 33.82%. Male sex, ethnicity, educational attainment, socioeconomic status, body mass index, waist circumference, tobacco use, and dyslipidemia were the prevalent risk factors (33). In Thailand, some studies applied the 3E2S principle to promote health in healthy older adults and to manage other NCDs, such as diabetes mellitus, stroke, and coronary artery diseases in older patients (15, 34). However, the present study suggests that only the first “E” (Eating) is significantly associated with blood pressure in the undiagnosed groups. All factors among 3E were separately investigated in the previous studies. A recent study by Wang and colleagues depicted that people with low exercise levels (spending more time sedentary) were more likely to have hypertension, while the levels of daily salt intake had no effect (35).

It was reported that 69% of Thais, to season food, used fish sauce or soy sauce, which have a sodium content of approximately 6,000 to 9,000 mg/100 mL of sauce (36). Food groups high in sodium comprised sompak (traditional fermented cabbage), instant noodles, processed meat (meatballs, ham, bacon, and sausages), sauces and pastes (fish sauce, oyster sauce, soy sauce, soybean paste, shrimp paste, and fish paste), and milk/soy drinks. Seasonings included salt, monosodium glutamate (MSG), chicken/pork seasoning powder, and other condiments (36, 37). Moreover, Thai people annually consume over 3 billion servings of instant noodles (≈50 servings/per person), which contain about 1,800 and 3,600 mg of salt in 100 grams of noodles (36). To our knowledge, the present study first reports the prevalence of seasoning powder among older Thai people (52 vs. 58% in normotensive and hypertensive individuals, respectively). According to nutrition labels, chicken and pork seasoning powder contains 32% MSG. According to Thongsepee and colleagues, MSG use contributes to oxidative stress, which might lead to hypertension and poor renal function (38). Therefore, this study suggests that health literacy on MSG use awareness should be disseminated among Thai people. With DASH (Dietary Approaches to Stop Hypertension), limiting alcohol, losing weight, and engaging in aerobic activity, a low-salt diet reduced blood pressure among hypertensive individuals by 14.2/7.4 mmHg (39). Reducing salt consumption is probably the most crucial hypotensive strategy, which includes avoiding processed foods, reading product labels for salt content, and flavoring food with herbs or spices (40).

It should be noted in the present study that the percentage of individuals having good eating habits was higher in the hypertensive group. This finding might be explained by an observation that hypertensive older adults tend to monitor their blood pressure by consuming less salt and having better self-reported behaviors (41). However, these self-reported behaviors did not align with their 24-h records of micronutrient intake – selenium, vitamin B6, and B12. We realized that the 24-h record is a relatively short timeframe. However, it has been previously proved that it was more practical than the 3-day record using the Institute of Nutrition, Mahidol University CALculation (INMUCAL) nutritional analysis program (42). Moreover, the 24-h and 3-day methods provided similar measured outcomes (macronutrients and micronutrients vs. blood chemistry). Secondly, INMUCAL has been compared to another nutrient-calculating program (CAN-Pro) from Korea, which shows no difference in average nutrient intake (43). Thirdly, the 24-h record and INMUCAL were successfully employed in older adults, reassuring their applicability in this population group (44). We propose that the 24-h record method is reliable and suitable for estimating micronutrient consumption levels.

Hypertensive individuals produce more reactive oxygen species (ROS) and have a reduced antioxidant defense system, worsening an oxidative stress cycle and bodily inflammatory process (45). Selenium was assumed to protect against hypertension since it is an essential trace element with antioxidant properties (46). Selenium is a crucial component of glutathione peroxidase, an enzyme that prevents lipid oxidation and the formation of atherosclerotic plaques by preventing vascular smooth muscle cell migration, blood clot formation, and platelet aggregation (47). A direct relationship between selenium and hypertension can be seen in Keshan disease (48). Keshan disease, also known as juvenile cardiomyopathy with pulmonary edema, is caused by a fusion of a mutant strain of the Coxsackie B virus and low selenium levels (49). Selenium supplements can reduce Keshan disease symptoms such as hypertension, heart failure, and pulmonary edema (48). However, over-supplementation or consuming a diet high in foods high in selenium can result in poisoning. In Venezuela, there have been reports of acute selenium poisoning caused by eating the fruit of the *Lecythis ollaria* species, which has a high selenium content (7–12 g selenium/kg of dry mass). The symptoms included hair loss, diarrhea, and emesis (50). Moreover, randomized trials incorporating selenium into multivitamin supplements have demonstrated a decrease in stomach cancer, stroke, and overall mortality. However, these interventions did not mitigate the risk of hypertension and cardiovascular risks (51). Therefore, implementing selenium supplements in older adults should be cautiously personalized.

A recent study in the United States showed that vitamin B6 and B12 were strongly related adversely to hypertension, indicating that these nutrients potentially have a protective impact on hypertension (52). Vitamin B6 (pyridoxine) may have mitigating effects on oxidative stress and inflammation (by preventing the cytokine outburst), control calcium ion (Ca^2+^) levels, raise a level of carnosine (a cardioprotective substance), and enhance immune system performance (53). The synthesis of interleukins and T cells critically depends on vitamin B6 (54). Consequently, a deficiency of it results in a reduction in immunity, which includes a rise in IL-4, a decrease in IL-2 production, and the development of serum antibodies. When chronic inflammation is present, there is an inverse association between vitamin B6, IL-6, and TNF-α levels (53). Low plasma vitamin B6 concentrations have been linked to adult hypertension (55). Aybak and co-workers found that taking 5 mg/kg/day of vitamin B6 for 4 weeks significantly reduced blood pressure by 14/10 mmHg (56). Meanwhile, vitamin B12 (cobalamin) may also serve as an anti-inflammatory agent by downregulating the transcription factor nuclear factor-kappa B (NF-κB), inhibiting nitric oxide synthase, and promoting oxidative phosphorylation (57). A direct measurement of plasma lipid peroxidation (8-isoprostane) showed a significant increase in oxidative stress in hypertensive individuals compared to healthy counterparts (58). These factors should be taken into account in our further study.

Other factors were crucial in machine learning prediction. A study by Wei and co-workers demonstrated that bean product consumption has reportedly been preventive for hypertension (59). This large cohort with more than 60,000 participants elaborately showed that Chinese people eating soybean products ≥125 g/day was adequate for reducing the incidence of hypertension by 27%. It has been proposed that soy nutrients such as proteins, isoflavones, phytosterols, and lecithin mitigate the risk of high blood pressure by enhancing endogenous nitric oxide synthesis, facilitating vasodilation, dispossessing free radicals, and reducing oxidative stress (60).

This study has limitations in the data collection process. First, serum biomarkers such as mineral and vitamin concentrations were helpful for clinical data analysis but were unavailable. Secondly, medication data were not collected, rendering food-drug interaction analysis. Thirdly, this study is preliminary and requires further external validation. Lastly, our findings were acquired from a relatively small group of older persons compared to the large cohort in the nationwide studies; thus, the result’s generalizability must be tested in other populations with caution. However, according to Vabalas et al., a sample size <1,000 is relatively small (61). Therefore, our total 5,288 participants (894 hypertensive cases and 4,394 normotensive individuals) can be considered a large sample size. Moreover, we also balanced the sample size in each class using the SMOTE method. Hence, we propose that our sample size has been justified. Further possible clinical application includes the development of a web-based screening tool for a UHTN risk group using an application processing interface.

In conclusion, this study demonstrated that based on binary logistic regression and supervised ML algorithms (random forest), high salt intake is a primary predictor of UHTN in older adults. On the other hand, consuming soybean and micronutrients, particularly vitamins B6, B12, and selenium, are preventive factors. Age should be a crucial confounder. We depict that ML is feasible to predict the UHTN based on 3E2S data in older adults. Extensive cohort studies should be performed to determine the exact mechanisms for modulating blood pressure in larger groups of populations.

## Data availability statement

The raw data supporting the conclusions of this article will be made available by the authors, without undue reservation.

## Ethics statement

The studies involving humans were approved by Maha Sarakham Provincial Public Health Office’s Ethical Review Committee for Human Research. The studies were conducted in accordance with the local legislation and institutional requirements. Written informed consent for participation in this study was provided by the participants’ legal guardians/next of kin.

## Author contributions

NT: Conceptualization, Funding acquisition, Investigation, Methodology, Project administration, Validation, Writing – original draft, Writing – review & editing. LN: Methodology, Resources, Supervision, Validation, Writing – original draft, Writing – review & editing. AB: Data curation, Formal analysis, Resources, Validation, Visualization, Writing – original draft, Writing – review & editing. AK: Data curation, Formal analysis, Software, Visualization, Writing – original draft, Writing – review & editing. KT: Conceptualization, Data curation, Formal analysis, Funding acquisition, Investigation, Methodology, Project administration, Resources, Supervision, Validation, Writing – original draft, Writing – review & editing.
