# Supplementary material for: Machine learning models identify micronutrient intake as predictors of undiagnosed hypertension among rural community-dwelling older adults in Thailand: a cross-sectional study
Source: Front Nutr. 2024 Jul 16;11:1411363. doi: 10.3389/fnut.2024.1411363 (PMC11286389; doi:10.3389/fnut.2024.1411363)
Supplement: Supplementary file 2 [file Table_2.DOCX]

**Cross-validation method**

We used Python 3.10.12 in Google Colab; case/control proportions were 894 hypertensive cases and 4,394 normotensive individuals (control) with 5-fold cross-validation as depicted below.


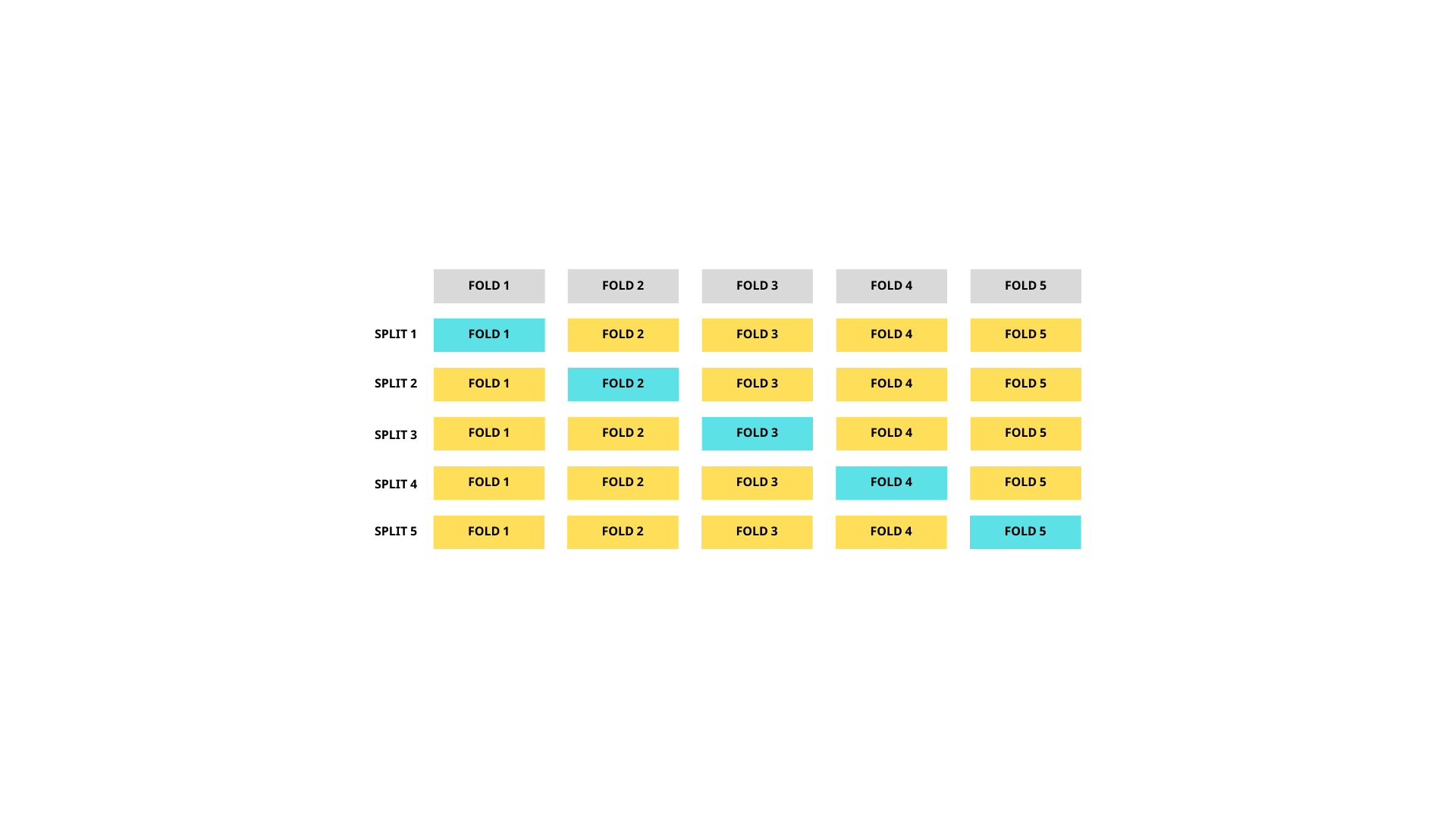


**Cross-validation scores:** [0.83549352, 0.81754736, 0.83632735, 0.7994012, 0.84431138]

**Average cross-validation score:** 0.8266161595055154

**Adjustments**

1. **Random Forest**

**Hyperparameters**

max_depth: [5,12,14,16,18,20], min_samples_leaf: [6,8,10,12,14,16], n_estimators:[10,25,30,50,70,100]

Choosing parameters max_depth,min_samples_leaf, and n_estimators in this range is important because choosing a higher value than this will cause overfitting, reducing the prediction model’s performance.

1. **SVM**

**Hyperparameters**

C: [0.1, 1, 10, 100, 1000], gamma: [1, 0.1, 0.01, 0.001, 0.0001], kernel: [‘linear’, ‘poly’, ‘rbf’, ‘sigmoid’]

Choosing parameters max_depth,min_samples_leaf, and n_estimators in this range is important because selecting a higher value than this will cause overfitting, reducing the prediction model’s performance.
