# Supplementary material for: Machine learning models identify micronutrient intake as predictors of undiagnosed hypertension among rural community-dwelling older adults in Thailand: a cross-sectional study
Source: Front Nutr. 2024 Jul 16;11:1411363. doi: 10.3389/fnut.2024.1411363 (PMC11286389; doi:10.3389/fnut.2024.1411363)
Supplement: Supplementary file 3 [file Data_Sheet_1.PDF]

STROBE Statement— Checklist of items that should be included in reports of ***cross-sectional studies***

	Item No.	Recommendation	Page No.	Relevant text from the manuscript
**Title and abstract**	1	(*a*) Indicate the study's design with a commonly used term in the title or the abstract	1	Machine learning models identify micronutrient intakes as predictors of undiagnosed hypertension among rural community-dwelling older adults in Thailand: A cross-sectional study
		(*b*) Provide in the abstract an informative and balanced summary of what was done and what was found	1	Objective: To develop a predictive model for undiagnosed hypertension (UHTN) in older adults based on five modifiable factors [eating behaviors, emotion, exercise, stopping smoking, and stopping drinking alcohol (3E2S) using machine learning (ML) algorithms.<br>Methods: The supervised ML models [random forest (RF), support vector machine (SVM), and extreme gradient boosting (XGB)] with SHapley Additive exPlanations (SHAP) prioritization and conventional statistics (χ^2 and generalized ordered logit (gologit) model were employed to predict UHTN from 5,288 health records of older adults from ten primary care hospitals in Thailand.<br>Results: The χ^2 analysis showed that eating behavior was the sole feature of UHTN occurrence. The gologit model revealed that taking food supplements/vitamins, using seasoning powder, and eating bean products were related to normotensive and hypertensive classifications. The RF, XGB, and SVM accuracy were 0.90, 0.89, and 0.57, respectively. The SHAP identified the importance of salt intake and food/vitamin supplements. Vitamin B6, B12, and selenium in the UHTN were lower than in the normotensive group.<br>Conclusion: ML indicates that salt intake and food/vitamin supplements are primary factors for UHTN classification in older adults.
**Introduction**				
Background/rationale	2	Explain the scientific background and rationale for the investigation being reported	3-5	"Hypertension is acknowledged as a significant risk factor for stroke and cardiovascular diseases worldwide (Boateng and Ampofo 2023)……………………………………………………………………………<br>These reports suggest alternative methods to predict UHTN besides the conventional statistical analyses.
Objectives	3	State specific objectives, including any prespecified hypotheses	5	Therefore, this present study aimed to identify the relationship between UHTN and 3E2S among older adults in Northeastern Thailand using conventional statistics (χ^2 analysis and generalized ordered logit models) and supervised ML models RF, SVM, XGB, and SHAP).
**Methods**				
Study design	4	Present key elements of study design early in the paper	5	Research design and participants<br>This descriptive cross-sectional study was performed on older adults from ten primary care hospitals in Northeastern Thailand.

Setting	5	Describe the setting, locations, and relevant dates, including periods of recruitment, exposure, follow-up, and data collection	5-6	Materials and Methods<br>Research design and participants and Data collection tools,
Participants	6	*Cross-sectional study*—Give the eligibility criteria, and the sources and methods of selection of participants	5	The inclusion criteria were age ≥ 60, not being diagnosed hypertensive, and a household registered with informed consent in four provinces. Older persons not present in person for the study were omitted. The participants with SBP < 140 mmHg were normotensive), while those ≥ 140 mmHg were hypertensive (Krzeminska et al. 2022; Williams et al. 2018).
Variables	7	Clearly define all outcomes, exposures, predictors, potential confounders, and effect modifiers. Give diagnostic criteria, if applicable		Data collection tools<br>There were two components to the data collection instruments used in the present study. Initially, information on gender, age, body mass index (BMI), marital status, and educational attainment was gathered. Because of the potential risks associated with disease, weakness, hospitalization, and falls, 75 was selected as the cut-off age (de Breij et al. 2021). In the second part, 3E2S data were collected. A structured questionnaire for consumption behaviors had 14 items of yes-no questions. A score of 11 was used as a cut-off point between good and poor consumption behaviors. A questionnaire on psychological stress was used as previously described (Tudpor et al. 2021). The physical activity score was 1 for those performing at least 150 minutes/week of moderate-intense exercise and 0 for those not following the WHO guidelines (World Health Organization 2010). Likewise, never or stop smoking tobacco and drinking alcohol were scored 1, while current smoking and drinking were scored 0. Three public health, psychiatry, and nursing specialists approved the instrument's content validity. The index of item-objective congruence (IOC) has verified the content validity. For the surveys, only items with IOC scores ≥ 0.5 were acceptable. The questionnaire included items that met the acceptable Cronbach's α ≥ 0.7 thresholds. The team of trained village health volunteers, nurses, public health practitioners, and physical therapists compiled the primary data in the area between April and August of 2021 through mobile phone apps and online forms.
Data sources/ measurement	8*	For each variable of interest, give sources of data and details of methods of assessment (measurement). Describe comparability of assessment methods if there is more than one group	5	Data collection tools<br>There were two components to the data collection instruments used in the present study. Initially, information on gender, age, body mass index (BMI), marital status, and educational attainment was gathered. Because of the potential risks associated with disease, weakness, hospitalization, and falls, 75 was selected as the cut-off age (de Breij et al. 2021). In the second part, 3E2S data were collected. A structured questionnaire for consumption behaviors had 14 items of yes-no questions. A score of 11 was used as a cut-off point between good and poor consumption behaviors. A questionnaire on psychological stress was used as previously described (Tudpor et al. 2021). The physical activity score was 1 for those performing at least 150 minutes/week of moderate-intense exercise and 0 for those not following the WHO guidelines (World Health Organization 2010). Likewise, never or stop smoking tobacco and drinking alcohol were scored 1, while current smoking and drinking

				were scored 0. Three public health, psychiatry, and nursing specialists approved the instrument's content validity. The index of item-objective congruence (IOC) has verified the content validity. For the surveys, only items with IOC scores ≥ 0.5 were acceptable. The questionnaire included items that met the acceptable Cronbach's α ≥ 0.7 thresholds. The team of trained village health volunteers, nurses, public health practitioners, and physical therapists compiled the primary data in the area between April and August of 2021 through mobile phone apps and online forms.
Bias	9	Describe any efforts to address potential sources of bias	-	None
Study size	10	Explain how the study size was arrived at	6	Sample size (n) was estimated using the formula as given: $n = \frac{Nz^2pq}{+d^2(N-1)+z^2pq}$ (Daniel and Cross 2018), where N represents the total number of the older adult population, which was 5,288, z = percentiles of the standard normal distribution corresponding to a 95% confidence level, which is 1.96, p = proportion of the older adults with undiagnosed hypertension, which was assumed to be 0.17, q = 1 − p, and d denotes margin of error = 5 at 95% confidence level. Therefore, using the formula, n = 5,288(1.96)2 × 0.17 × 0.83/$(0.05)^2$(5,288 - 1) + $(1.96)^2$ × 0.17 × 0.83 = 208.32 ~209.
Quantitative variables	11	Explain how quantitative variables were handled in the analyses. If applicable, describe which groupings were chosen and why	8	Machine learning models for hypertensive risk prediction and Statistical analyses
Statistical methods	12	(*a*) Describe all statistical methods, including those used to control for confounding	8	Statistical analyses<br>Continuous data are expressed as mean and standard deviation. The normality of sample data was tested using the Kolmogorov–Smirnov test. Two means of independent non-normally distributed samples were analyzed using the Mann-Whitney U test. Categorical data are expressed as frequency and percentage. For the univariate analysis, relationships between SBP, demographic variables, and 3E2E variables were evaluated and compared using χ^2 test. The results showed that there was minimal multicollinearity among the variables. Multivariable analysis of the generalized ordered logit models was performed to investigate the simultaneous effect of multiple factors on a dichotomous outcome using SPSS software version 18.
		(*b*) Describe any methods used to examine subgroups and interactions	-	Not relevant.
		(*c*) Explain how missing data were addressed	-	Not applicable.
		(*d*) *Cross-sectional study*—If applicable, describe analytical methods taking account of sampling strategy	5	Research design and participants<br>This descriptive cross-sectional study was performed on 5,288 health records of non-hypertensive older adults from ten primary care hospitals in Northeastern Thailand.
		(*e*) Describe any sensitivity analyses		None.

**Results**				
Participants	13*	(a) Report numbers of individuals at each stage of study—eg numbers potentially eligible, examined for eligibility, confirmed eligible, included in the study, completing follow-up, and analysed	5	Research design and participants<br>This descriptive cross-sectional study was performed on 5,288 health records of non-hypertensive older adults from ten primary care hospitals in Northeastern Thailand.<br>Sample size (n) was estimated using the formula as given: $n = \frac{Nz^2pq}{+d^2(N-1)+z^2pq}$ (Daniel and Cross 2018), where N represents the total number of the older adult population, which was 5,288, z = percentiles of the standard normal distribution corresponding to a 95% confidence level, which is 1.96, p = proportion of the older adults with undiagnosed hypertension, which was assumed to be 0.17, q = 1 − p, and d denotes margin of error = 5 at 95% confidence level. Therefore, using the formula, n = 5,288(1.96)2 × 0.17 × 0.83/$(0.05)^2$(5,288 - 1) + $(1.96)^2$ × 0.17 × 0.83 = 208.32 ~209.
		(b) Give reasons for non-participation at each stage	-	Not applicable.
		(c) Consider use of a flow diagram	-	Not applicable.
Descriptive data	14*	(a) Give characteristics of study participants (eg demographic, clinical, social) and information on exposures and potential confounders	5	Research design and participants<br>This descriptive cross-sectional study was performed on 5,288 health records of non-hypertensive older adults from ten primary care hospitals in Northeastern Thailand. This subproject was a part of the Ministry of Digital Economy and Society's Office of the National Digital Economy and Society Commission project, "Development of Mobile Application of Database of Older Persons Using Geographic Information System (GIS) to Detect and Analyze Risks of Chronic Diseases, Quality of Life, and Mental Illness by Village Health Volunteers in 7th Regional Health Office Territory." Each protocol followed the Declaration of Helsinki and was authorized by the Maha Sarakham Provincial Public Health Office's Ethical Review Committee for Human Research (No. 6/2564). The study complies with the recommendations of the Committee on Publication Ethics (COPE) and the International Committee of Medical Journal Editors (ICMJE) for ethics. Each participant provided written informed permission on an official document. The inclusion criteria were age ≥ 60, not being diagnosed hypertensive, and a household registered with informed consent in four provinces. Older persons not present in person for the study were omitted. The participants with SBP < 140 mmHg were normotensive), while those ≥ 140 mmHg were hypertensive (Krzeminska et al. 2022; Williams et al. 2018).
		(b) Indicate number of participants with missing data for each variable of interest	5-7	5,288 for machine learning and 209 for micronutrient analysis.
		Cross-sectional study—Report numbers of outcome events or summary measures	5-7	5,288 for machine learning and 209 for micronutrient analysis.

Main results	16	(*a*) Give unadjusted estimates and, if applicable, confounder-adjusted estimates and their precision (eg, 95% confidence interval). Make clear which confounders were adjusted for and why they were included	Table 3	See Table 3. Multivariable analysis of the generalized ordered logit models for age and eating behavior levels
Other analyses	17	Report other analyses done—eg analyses of subgroups and interactions, and sensitivity analyses	-	None.
**Discussion**				
Key results	18	Summarise key results with reference to study objectives	10-15	This study shows that consumption behaviors are predictors that classify blood pressure as normotension and hypertension in community-dwelling older adults. The following findings support our statement. First, among 3E2S, only consumption behaviors are significantly associated with the difference in systolic blood pressure. Secondly, using salt-containing seasoning powder is a risk factor for hypertension, while taking food supplements/vitamins is a preventive factor for hypertension. Risk and preventive factors for hypertension are different and overlap among countries. In the United Kingdom, the National Institute for Health and Clinical Excellence (NICE) has developed a practical clinical guideline on hypertension that encourages healthy, calorie-controlled diets, regular aerobic exercise, and reducing alcohol, salt, and tobacco use in overweight individuals with elevated blood pressure (Nicoll and Henein 2010). In Cameroon, the prevalence of high blood pressure in adults was 19.8%. Age, alcohol use, and a sedentary lifestyle were found to be separate causes of hypertension (Princewel et al. 2019). In the Chinese population, HTN incidence was 28%. Risk variables for hypertension were body mass index, level of physical activity, use of alcohol and tobacco, once-weekly meat consumption, family history, and daily salt intake (Zhang et al. 2021). A study in Bangladesh showed that four primary modifiable risk factors for hypertension are obesity, dyslipidemia, tobacco use, and excessive salt intake (Paul et al. 2020). Antihypertensive medications and these factors work together to prevent and treat hypertension. In India, after controlling for other risk variables, it was discovered that diabetes, smoking, obesity, and inactivity were all substantial risk factors for hypertension (Pilakkadavath and Shaffi 2016). The estimated prevalence of hypertension in Southeast Asians was 33.82%. Male gender, ethnicity, educational attainment, socioeconomic status, body mass index, waist circumference, tobacco use, and dyslipidemia were the prevalent risk factors (Mohammed Nawi et al. 2021). In Thailand, Visanuyothin and co-workers demonstrated the effectiveness of 3E2S activities combined with regularly taking antihypertensive medications and recognizing acute episodes to control blood pressure in primary care settings (Visanuyothin et al. 2018). Another study applied the 3E2S principle to promote health in healthy older adults (Visuttranukul et al. 2021). Recently, the 3E2S principle has been utilized to manage other NCDs, such as diabetes mellitus, stroke, and coronary artery diseases in older adults (Pitchalard et al. 2022). However, the

				present study suggests that only the first “E” (Eating) is significantly associated with blood pressure in the undiagnosed groups. All factors among 3E were separately investigated in the previous studies. A recent study by Wang and colleagues depicted that people with low exercise levels (spending more time sedentary) were more likely to have hypertension, while the levels of daily salt intake had no effect (Wang et al. 2022). Meanwhile, psychological stress was linked to high lipid-rich food consumption and subsequent hypertension (Dalmazo et al. 2019).<br>It was reported that 69% of Thais, to season food, used fish sauce or soy sauce, which have a sodium content of approximately 6,000 to 9,000 mg/100 ml of sauce (Chailek et al. 2023). Food groups high in sodium comprised sompak (traditional fermented cabbage), instant noodles, processed meat (meatballs, ham, bacon, and sausages), sauces and pastes (fish sauce, oyster sauce, soy sauce, soybean paste, shrimp paste, and fish paste), and milk/soy drinks. Seasonings included salt, monosodium glutamate (MSG), chicken/pork seasoning powder, and other condiments (Chailek et al. 2023; Lee et al. 2013b). Moreover, Thai people annually consume over 3 billion servings of instant noodles (≈50 servings/per person), which contain about 1,800 and 3,600 mg of salt in 100 grams of noodles (Chailek et al. 2023). To our knowledge, the present study first reports the prevalence of seasoning powder among older Thai people (52 vs 58% in normotensive and hypertensive individuals, respectively). According to nutrition labels, chicken and pork seasoning powder contains 32% MSG. According to Thongsepee and colleagues, MSG use contributes to oxidative stress, which might lead to hypertension and poor renal function (Thongsepee et al. 2022). Therefore, this study suggests that health literacy on MSG use awareness should be disseminated among Thai people. With DASH (Dietary Approaches to Stop Hypertension), limiting alcohol, losing weight, and engaging in aerobic activity, a low-salt diet reduced blood pressure among hypertensive individuals by 14.2/7.4 mmHg (Appel et al. 2003). Reducing salt consumption is probably the most crucial hypotensive strategy, which includes avoiding processed foods, reading product labels for salt content, and flavoring food with herbs or spices (Mahtani 2009).<br>Reactive oxygen species (ROS) overproduction and a compromised antioxidant system accelerate the growth of endothelial dysfunction, inflammation, and enhanced vascular contractility, which remodels vascular architecture (Stanley et al. 2019). Hypertensive individuals produce more ROS and have a reduced antioxidant defense system, worsening an oxidative stress cycle and bodily inflammatory process (Touyz et al. 2020). Selenium was assumed to protect against hypertension since it is an essential trace element with antioxidant properties (Kuruppu et al. 2014). Selenium is a crucial component of glutathione peroxidase, an enzyme that prevents lipid oxidation and the formation of atherosclerotic plaques by preventing vascular smooth muscle cell migration, blood clot formation, and platelet aggregation (Dabravolski et al. 2023). A direct relationship between selenium and

				hypertension can be seen in Keshan disease (Boosalis 2008). Keshan disease, also known as juvenile cardiomyopathy with pulmonary edema, is caused by a fusion of a mutant strain of the Coxsackie B virus and low selenium levels (Loscalzo 2014). Selenium supplements can reduce Keshan disease symptoms such as hypertension, heart failure, and pulmonary edema (Boosalis 2008). However, over-supplementation or consuming a diet high in foods high in selenium can result in poisoning. In Venezuela, there have been reports of acute selenium poisoning caused by eating the fruit of the Lecythis ollaria species, which has a high selenium content (7–12 g selenium/kg of dry mass). The symptoms included hair loss, diarrhea, and emesis (Fordyce 2007). Moreover, randomized trials incorporating selenium into multivitamin supplements have demonstrated a decrease in stomach cancer, stroke, and overall mortality. However, these interventions did not mitigate the risk of hypertension and cardiovascular risks (Huang et al. 2006). Therefore, implementing selenium supplements in older adults should be cautiously personalized.<br>A recent study in the United States showed that vitamin B6 and B12 were strongly related adversely to hypertension, indicating that these nutrients potentially have a protective impact on hypertension (Xiong et al. 2023). Vitamin B6 (pyridoxine) may have mitigating effects on oxidative stress and inflammation (by preventing the cytokine outburst), control calcium ion (Ca2+) levels, raise a level of carnosine (a cardioprotective substance), and enhance immune system performance (Stach et al. 2021). The synthesis of interleukins and T cells critically depends on vitamin B6 (Qian et al. 2017). Consequently, a deficiency of it results in a reduction in immunity, which includes a rise in IL-4, a decrease in IL-2 production, and the development of serum antibodies. When chronic inflammation is present, there is an inverse association between vitamin B6, IL-6, and TNF-α levels (Stach et al. 2021). Low plasma vitamin B6 concentrations have been linked to adult hypertension (Houston 2014). Aybak and co-workers found that taking 5 mg/kg/day of vitamin B6 for four weeks significantly reduced blood pressure by 14/10 mmHg (Aybak et al. 1995). Meanwhile, vitamin B12 (cobalamin) may also serve as an anti-inflammatory agent by downregulating the transcription factor nuclear factor-kappa B (NF-□B), inhibiting nitric oxide synthase, and promoting oxidative phosphorylation. (Lee et al. 2016). A direct measurement of plasma lipid peroxidation (8-isoprostane) showed a significant increase in oxidative stress in hypertensive individuals compared to healthy counterparts (Rodrigo et al. 2007). These factors should be taken into account in our further study.<br>Other factors were crucial in the Chi-square analysis, but not machine learning prediction. Breakfast skippers reportedly skip breakfast daily and eat less than three times a week. It has been recently established that hypertension is associated with skipping breakfast (Li et al. 2022). Physiologically, skipping breakfast can cause hypertension in several ways. First, missing breakfast can lead to appetite changes

				and overeating later in the day, resulting in insulin resistance and overweight (Ma et al. 2020). The links between hypertension, insulin resistance, and overweight are well-established (Mao et al. 2022). Secondly, skipping breakfast can be behaviorally indicated by unhealthy lifestyles, such as poor food consumption habits, physical inactivity, and inconsistent sleep patterns (Faris et al. 2022; Kohyama 2021). These behaviors could elevate the person's risk of hypertension. In addition, a study by Wei and co-workers demonstrated that bean product consumption has reportedly been preventive for hypertension (Wei et al. 2020). This large cohort with more than 60,000 participants elaborately showed that Chinese people eating soybean products $\geq$ 125 g/day was adequate for reducing the incidence of hypertension by 27%. It has been proposed that soy nutrients such as proteins, isoflavones, phytosterols, and lecithin mitigate the risk of high blood pressure by enhancing endogenous nitric oxide synthesis, facilitating vasodilation, dispossessing free radicals, and reducing oxidative stress (Ramdath et al. 2017).
Limitations	19	Discuss limitations of the study, taking into account sources of potential bias or imprecision. Discuss both direction and magnitude of any potential bias	15	This study has limitations in the data collection process. First, serum biomarkers such as mineral and vitamin concentrations were helpful for clinical data analysis but were unavailable. Secondly, medication data were not collected, rendering food-drug interaction analysis.
Interpretation	20	Give a cautious overall interpretation of results considering objectives, limitations, multiplicity of analyses, results from similar studies, and other relevant evidence	15	In conclusion, this study demonstrated that based on supervised ML algorithms (random forest), high salt intake is a primary predictor of UHTN in older adults. On the other hand, micronutrients, particularly vitamins B6, B12, and selenium, are preventive factors. Psychological stress, BMI, age, breakfast, and consuming bean products should be crucial confounders. We depict that ML is feasible to predict the UHTN based on 3E2S data in older adults. Extensive cohort studies should be performed to determine the exact mechanisms for modulating blood pressure in larger groups of populations.
Generalisability	21	Discuss the generalisability (external validity) of the study results	15	Lastly, our findings were acquired from a small group of older persons; thus, the result's generalizability must be tested in other populations with caution.
**Other information**				
Funding	22	Give the source of funding and the role of the funders for the present study and, if applicable, for the original study on which the present article is based	15	This research project was financially supported by Mahasarakham University (grant no. 6607052/2566).

*Give information separately for cases and controls in case-control studies and, if applicable, for exposed and unexposed groups in cohort and cross-sectional studies.
